# To the Editor of the Medico-Chirurgical Review

**Published:** 1831

**Authors:** 


### Periscope;

OR,

### CIRCUMSPECTIVE REVIEW.

" Ore trahit quodcunque potest, atque addit acervo."

I.

To the Editor of the Medico-chirurgical Review.

*Apothecaries Hall,*
*April* 14*th*, 1831.

Sir,

Numerous applications continuing to be made by gentlemen in almost every department of medical science, desiring to be recognised as lecturers by the Court of Examiners of the Society of Apothecaries, the Court feel anxious, in order to save the time now necessarily expended in correspondence with each successive applicant, that the Rules which the Court have laid down for their own guidance in the recognition of the various courses of lectures required by them should become generally known to the profession: they have therefore desired me to transmit these rules to your Journal, with a request that you will be kind enough to give them an early insertion.

I have the honor to be, Sir,
Your obedient Servant,
John Watson,
Secretary to the Court of Examiners.

*Rules to be observed in the Recognition of Lecturers, extracted from the Minutes of the Court of Examiners, dated Nov.* 18*th*, 1830.

Resolved,

1. That any person being a Member of the Court of Examiners shall not be recognised as a lecturer on any branch of medical science.
2. That the Court will not recognise any new teacher who may give lectures on more than two branches of medical science; nor will they sanction a teacher already recognised in giving lectures on any *third* branch of the science, if already he gives lectures on *two*.
3. That the Court will not recognise a teacher until he has given a public course of lectures on the subject he purposes to teach; but if, after such preliminary course of lectures, the teacher should be recognised, the student's certificate of attendance on that course will be received.
4. That the Court will not recognise a teacher until he has produced very satisfactory testimonials of his attainments in the science he purposes to teach, and also of his ability as a teacher of it, from persons of acknowledged talents and distinguished acquirements in the particular branch of science in question.
5. That satisfactory assurance shall also be given that the teacher is in possession of the means requisite for the full illustration of his lectures, viz. that he has, if lecturing on Chemistry, a laboratory and competent apparatus: on Materia Medica, a museum sufficiently extensive: on Anatomy and Physiology, a museum sufficiently well furnished with preparations, and the means of procuring recent subjects for demonstration: on Botany, a hortus siccus, plates or drawings, and the means of procuring fresh specimens: on Midwifery, a museum and such an appointment in a public midwifery institution as may enable him to give his pupils practical instructions.

6. That the Lecturer on *the Principles and Practice of Medicine* must be, if he lectures in London, or within seven miles thereof, a Fellow, Candidate, or Licentiate of the Royal College of Physicians of London: and if he lectures beyond seven miles from London, and should not be thus qualified, he must be a Graduated Doctor of Medicine of a British University of four years standing, (unless previously to his graduation he had been for four years a Licentiate of this Court.)
7. That the *Lecturer on Materia Medica and Therapeutics* must be a Fellow, Candidate, or Licentiate of the Royal College of Physicians of London, a Graduated Doctor of Medicine of a British University of four years standing, (unless previously to his graduation he had been for the same length of time a Licentiate of this Court) or he must be a Licentiate of this Court of four years standing.
8. That the Lecturer on *Anatomy and Physiology* must either be recognised by the Royal College of Surgeons, of London, or must be a member of that College of four years standing.
9. That the *Demonstrator of Anatomy* must either be recognized by the Royal College of Surgeons of London, or must be a Member of that College.

---

We give the Court of Examiners all credit for good intentions in the curricula which they issue, from time to time, for the guidance of the aspirants in medicine; but we confess that one or two of the foregoing regulations appear to us to indicate a strong disposition in the Court to assume unlimited power over the teachers as well as the pupils. Lecturers of 20 years standing, must now, in fact, present themselves at Apothecaries' Hall (by the 4th section) and convince the Court of their capacity to teach. Dr. Clutterbuck, Dr. Agar, Dr. Hue, Dr. Chambers, &c. must all repair to the Hall! In the 5th Section, there is a range given for every species of tyranny and oppression if the Court were that way inclined, in consequence of the indefinite terms used. Thus, in chemistry, the laboratory must have a *competent* apparatus. The anatomist must have a *sufficiently* well furnished museum, and so on. Now it is clear that the mere opinion, the caprice, or even the prejudice of that member of the Court who examines the domicile of the lecturer, may fix the *sufficiency* high or low, according to his humour that day! If laws are made, and meant to be enforced, they should be definite, and not left wide open for every interpretation which the judge choses to give them.

